# The mitochondrial genome of *Erronea caurica* (Cypraeidae)

**DOI:** 10.1080/23802359.2020.1860693

**Published:** 2021-02-08

**Authors:** Lirong Bai, Dahui Yu, Xueyu Yan, Jiawen Liu, Haoxin Jiang, Zhiying Zhao, Xia Liang

**Affiliations:** a Guangxi Key Laboratory of Beibu Gulf Marine Biodiversity Conservation, Beibu Gulf University, Qinzhou, PR China; b Hainan Academy of Ocean and Fisheries Sciences, Haikou, PR China

**Keywords:** Mitochondrial genome, *Erronea caurica*, phylogenetic tree

## Abstract

The mitochondrial genome of *Erronea caurica* from the South China Sea has been determined (GenBank Accession No. MT522622), which was the second report of mitochondrial genome in the superfamily Cypraeoidea. It is 16,053 bp long and consists of 21 tRNA genes, 2 rRNA genes, 13 protein-coding genes, and 1 control region. As previously reported mitochondrial genome in Cypraeoidea, all protein-coding genes of *E. caurica* use a typical start codon (ATN) and a complete stop codon (TAA or TAG). Phylogenetic tree demonstrated that *E. caurica* belongs to the family Cypraeoidea and closer to the superfamily Tonnoidea.

*Erronea caurica* is a species of sea snail, a cowry, a marine gastropod mollusk in the family Cypraeidae. It can reach up to 5 cm in length with thick-edged cowry shell shape that elongated and a light brown or yellowish basic color, and a pinkish underside and brown spots on the edge. It occurs in large numbers over the majority of the tropical Indo-Pacific region, except Hawaii and southeastern Polynesia. As one of the most well-known sea snails for the commercially valuable shells, the decreased collection by people significantly affected its abundance (Newton et al. 1993). For the conservation and biodiversity study, we sequenced and assembled the mitochondrial genome of *E. caurica*. It was the second report of mitochondrial genome in the superfamily Cypraeoidea, with the first reported mitochondrial of this superfamily was *Cypraea tigris* (Pu et al. 2019).

Our specimens used in this study were collected from the South China Sea (19°29′42″N, 112°24′32″E) and stored in Guangxi Key Laboratory of Beibu Gulf Marine Biodiversity Conservation, Beibu Gulf University (specimen voucher number: BGU0328). Total DNA was isolated from muscle samples of *E. caurica* using QIAGEN (Germany) DNA Extraction Kit and then sequenced on Illumina Solexa HiSeq2000. A final of 10 Gb raw sequencing data (after initial quality score) with a paired end reads length of 2 × 150 bp were produced and used for multiple runs of mitochondrial genome assembly. The mitochondrial genome was assembled using CLC Genomics Workbench (Version 10.1) with *Cypraea tigris* (Pu et al. 2019) as a reference sequence and then annotated with GeSeq (Tillich et al. 2017).

The complete mitochondrial genome of *E. caurica* is 16,053 bp in length (GenBank Accession No. MT522622). The total base content of *E. caurica* mitogenome was 28.75% A, 16.10% G, 36.78% T, and 14.21% C. and a total GC content of 30.31%. It consists of 13 protein-coding genes, 2 rRNA genes, 21 tRNA genes, and 1 control region (D-loop). Similar to previously reported *C. tigris* mitochondrial genome (Pu et al. 2019), most of the genes were encoded on the heavy strand except for seven tRNA genes (tRNA-Met, -Tyr, -Cys, -Trp, -Gly, -Glu, and tRNA-Thr). All the 13 protein-coding genes of *E. caurica* mitogenome use the typical initiation codon ATN (10 genes use ATG; COX3 and ND4L use ATA; ND4 uses ATT); all of them end with a complete stop codon (9 genes use TAA; 4 genes use TAG). The 12S rRNA is 972 bp and located between tRNA-Glu and tRNA-Val, and the 16S rRNA is 1388 bp, located between tRNA-Val and tRNA-Leu. The 21 tRNA genes were varied from 61 to 72 bp in length. The control region is located between tRNA-Phe and COX3 with a length of 911 bp.

For phylogenetic analysis, we downloaded Littorinimorpha mitogenomes available in GenBank using the maximum-likelihood (ML) method with 1000 bootstrap replicates and General Time Reversible (GTR) substitution model assisted with Gamma distributed with Invariant sites (G + I) rates. The phylogenetic tree (Figure 1) demonstrated that that *E. caurica* belongs to Cypraeoidea and closer to the superfamily Tonnoidea, and further clarified the phylogenetic relationships of the superfamily in Littorinimorpha compared with the previous work by single mitochondrial genes (Meyer 2003; Sun et al. 2012; Pu et al., 2019).

**Figure 1.**
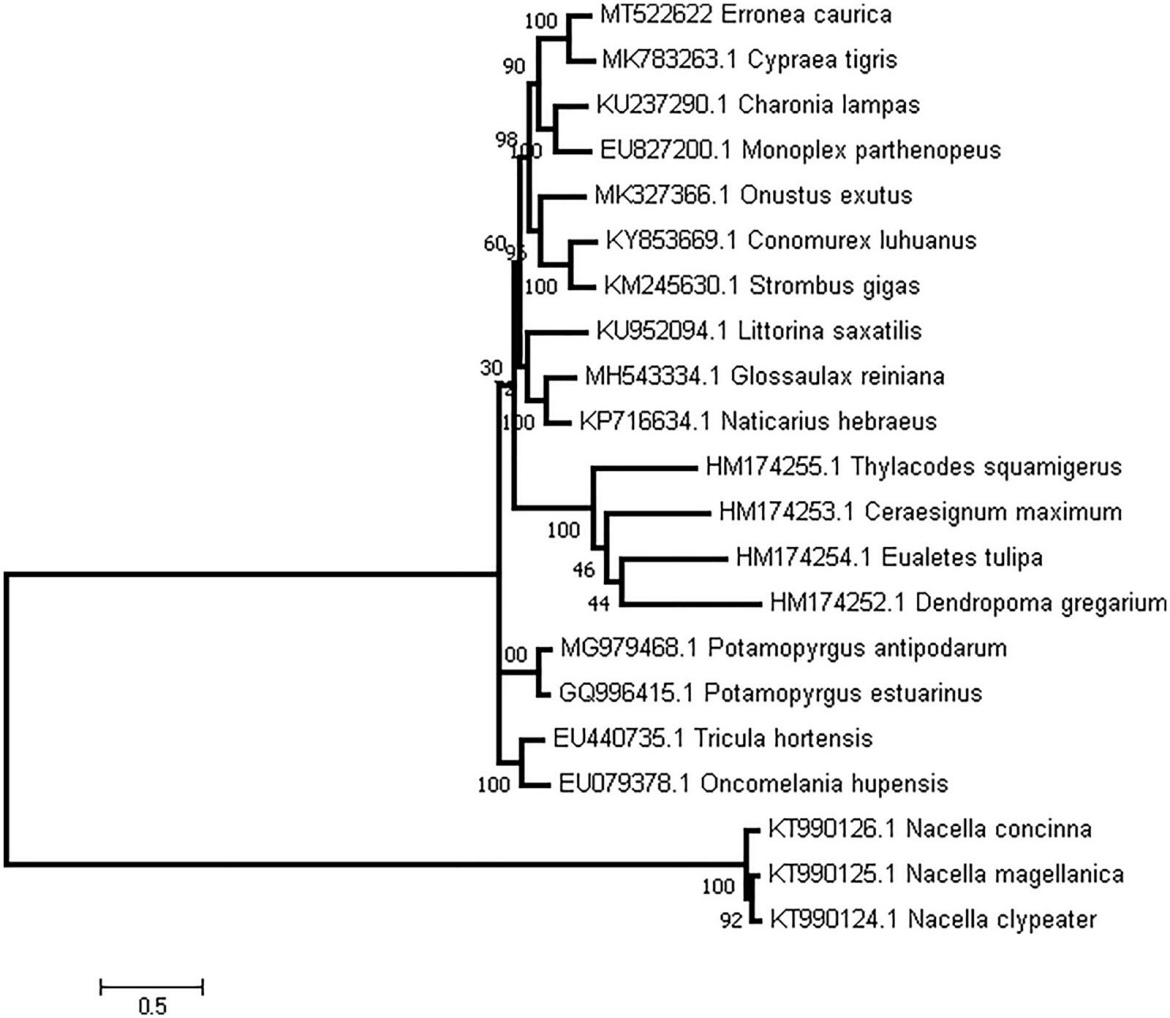
A maximum-likelihood tree illustrates the phylogenetic position of *E. caurica* among other species.

## Data Availability

The data that support the findings of this study are openly available in GenBank of NCBI at https://www.ncbi.nlm.nih.gov, reference number MT522622.
